# An estimation method for inference of gene regulatory net-work using Bayesian network with uniting of partial problems

**DOI:** 10.1186/1471-2164-13-S1-S12

**Published:** 2012-01-17

**Authors:** Yukito Watanabe, Shigeto Seno, Yoichi Takenaka, Hideo Matsuda

**Affiliations:** 1 Department of Bioinformatic Engineering, Graduate School of Information Science and Technology, Osaka University, Osaka, Japan

## Abstract

**Background:**

Bayesian networks (BNs) have been widely used to estimate gene regulatory networks. Many BN methods have been developed to estimate networks from microarray data. However, two serious problems reduce the effectiveness of current BN methods. The first problem is that BN-based methods require huge computational time to estimate large-scale networks. The second is that the estimated network cannot have cyclic structures, even if the actual network has such structures.

**Results:**

In this paper, we present a novel BN-based deterministic method with reduced computational time that allows cyclic structures. Our approach generates all the combinational triplets of genes, estimates networks of the triplets by BN, and unites the networks into a single network containing all genes. This method decreases the search space of predicting gene regulatory networks without degrading the solution accuracy compared with the greedy hill climbing (GHC) method. The order of computational time is the cube of number of genes. In addition, the network estimated by our method can include cyclic structures.

**Conclusions:**

We verified the effectiveness of the proposed method for all known gene regulatory networks and their expression profiles. The results demonstrate that this approach can predict regulatory networks with reduced computational time without degrading the solution accuracy compared with the GHC method.

## Background

Finding gene regulations is an important objective of systems biology [1,2]. Causal gene regulatory interactions are widely described using gene regulatory networks. Estimating gene regulatory networks can help reveal complicated regulations.

Recently, microarray [3,4] has rapidly produced a wealth of information about gene expression activities. The volume of data necessitates computational methods to identify and analyze the underlying gene regulatory networks [5]. A number of analytical methods have been proposed to estimate gene regulatory networks from gene expression profiles. Boolean networks, graphical Gaussian models (GGM), differential equation models, and Bayesian networks (BNs) are widely used models.

A Boolean network is a discrete dynamical network [6,7]. In a Boolean network, the state of a gene is represented by a Boolean variable (ON or OFF) and interactions between the genes are represented by Boolean functions that determine the state of a gene on the basis of the states of certain other genes. Hence, continuous gene expression data must be transformed into binary data before a Boolean network can be estimated, and much information is lost in this binary encoding. As gene expression cannot be described adequately by only two states, Boolean networks are limited by their definition.

A GGM is an undirected probabilistic graphical model [8]. This model allows the identification of conditional independence relations among the nodes under the assumption of a multivariate Gaussian distribution of the data. In a GGM, regulations between genes are estimated by calculating the correlation between pairs of variables. Therefore, the GGM does not identify the direction of regulatory relationships between two genes, but rather only calculates the correlations between their gene expression data.

A differential equation model describes gene expression changes as a function of the expression of other genes and environmental factors [9-11]. Their flexibility allows the complex relations among components to be described. In a differential equation model, a gene regulation is described as the function of several gene expression levels. When the input data includes experimental noise, this model cannot estimate the gene regulatory network accurately. Also, if there is not sufficient data input, overfitting occurs.

BN is a graphical model for representing probabilistic relationships among a set of random variables [12-16]. These relationships are encoded in the structure of a directed acyclic graph whose nodes are the random variables. The relationships between the variables are described by a joint probability distribution. In a BN, causal interactions between more than three genes can be estimated. BN has advantages over the above models in applications where BN deals better with the experimental noise.

Using a BN, it is hard to estimate a large-scale network because the search space grows exponentially as the number of genes increases. Therefore, overcoming this problem has been the focus of much research. The proposed solutions to this problem can be divided into three types. The first type limits the number of estimated genes. Even when estimating a large-scale network, part of the network is often attracted. The second type parallelizes the estimation by supercomputer or other high-performance computer. Effective parallelizing makes it possible to estimate large-scale networks. The third type improve the algorithm itself. These methods reduce computational time and estimate the network by a heuristic.

An example of the first type of solution is proposed by Peña *et al. *[17]. This method overcomes the problem of the user having to decide in advance which genes are included in or excluded from the learning process. The method receives a seed gene *S *and a positive integer *R *from the user, and returns a BN. It starts the BN from *S *genes, then adds the parents and children of all the genes in the BN *R + 1 *times, and prunes some genes. In this way, the user avoids deciding in advance which genes to include.

A solution of the second type proposed by Tamada *et al. *[18] can estimate gene regulatory networks consisting of more than 20,000 genes from gene expression data. The method uses a supercomputer, and it is massively parallelized. It repeatedly estimates subnetworks by hill climbing in parallel for genes selected by *neighbor node sampling*. The method high-handedly overcomes the problem of the BN by using the supercomputer. Even if a supercomputer can effectively provide a large-scale network, an estimation method designed to run on a workstation is also required.

A solution of the third type for estimating gene regulatory networks was implemented by Bøttcher *et al. *[19]: the greedy hill climbing (GHC) method. By comparing networks that differ only by a single directed edge, either added, removed, or reversed, a GHC method can estimate networks of larger scale than a search of all possible networks and do so on a workstation rather than a supercomputer, thus overcoming two problems at once. However, the estimation accuracy of this method is not high, because the method tends to produce only local optimal solutions.

In this paper, we present a novel BN-based deterministic method with reduced computational time to overcome the above-mentioned problems. The proposed method can estimate a network as large-scale as those estimated by the GHC method, run on a workstation, and estimate more accurately than the GHC method. We take another approach to estimate more accurately than the GHC method. First, our method generates all the combinational subsets with three genes. Then, we estimate all possible networks for each subset using the BN method and unite the networks into a single network including all genes. This approach enables us to estimate more accurately for the same computational time than the GHC method.

In order to verify the effectiveness of the proposed method, we perform two experiments, to evaluate scalability and accuracy: i.e., one to verify the proposed method can estimate networks as large-scale as those estimated by the GHC method, and one to verify it can estimate more accurately than the GHC method. These experiments are performed using randomly sampled genes. In addition, we conduct a third experiment to confirm that our method outperforms the GHC method using real data.

## Results

### Bayesian networks

Let *D = *(*V, E*) be a directed acyclic graph (DAG), where *V *is a finite set of nodes and *E *is a finite set of directed edges between the nodes [19]. The DAG defines the structure of the BN.

Each node *v *∈ *V *in the graph corresponds to a random variable *x*_*v*_. The set of variables associated with the graph *D *is then *X = *{*x*_*v*_}. Often we do not distinguish between a variable *x*_*v *_and the corresponding node *v. *To each node *v *with parents *pa*(*v*), a local probability distribution, *p*(*x*_*v*_*|x*_*pa*__(__*v*__)_), is attached. The set of local probability distributions for all variables in the network is *P*. A BN for a set of random variables *X *is the pair (*D,P*). Directed edges in *D *encode conditional dependencies between the random variables *X *through the factorization of the joint probability distribution.

(1) $$p\left(x\right)=\underset{v\in V}{\prod }p\left(x_{v}|x_{pa\left(v\right)}\right).$$

As a measure of how well a DAG D represents the conditional dependencies between the random variables, we use the relative probability

(2) p(D,d)=p(d|D)p(D),

and refer to it as a network score, where *d *is data and *p*(*d|D*) is called the likelihood of *D*.

The log network score contribution of a node is evaluated whenever the node is learned. The log network score *N*(*D*) is given by

(3) N(D)= logp(D,d).

The number of possible DAGs grows exponentially with the number of nodes, and the problem of identifying the network with the highest score is NP-hard. If the number of random variables in a network is large, it is not computationally possible to calculate the network score for all possible DAGs. For these situations, the search strategy *GHC method *is implemented.

The GHC method is as follows.

1. Select an initial DAG *D*_0 _randomly from which to start the search.

2. Calculate the Bayes scores of *D*_0 _and all possible networks that differ by only one directed edge, that is, an edge is added to *D*_0_, an edge in *D*_0 _is deleted, or the direction of an edge in *D*_0 _is reversed.

3. Among all these networks, select the one that increases the Bayes score the most.

4. If the Bayes score was not improved, stop the search. Otherwise, make the select network *D*_0 _and repeat from step 2.

In the GHC method, we can limit the maximum number of these steps in the search algorithm. Also, the search algorithm can restart an arbitrary number of times. More details on the parameter setting will be described later in this paper.

### Methods

**Figure 1:**
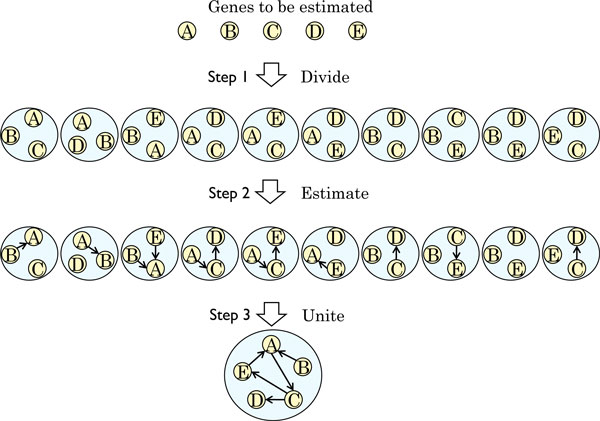
**Conceptual representation of our approach.** Yellow circles represent genes. Blue circles represent partial problems. Small directed edges represent regulatory relationships between genes. Large directed edges represent the flow of the method.

We propose a new method to estimate a gene regulatory network with reduced computational time. The proposed method is composed of three steps: dividing the whole problem into partial problems, estimating gene regulatory networks of partial problems, and uniting the estimated networks. In this section, we describe our BN-based method using the analysis of a set of expression data as an example. This example includes five genes *V *= {*v*_*i*_|1 ≤ *i *≤ 5}. A conceptual representation of our approach is presented in Figure 1. We call a search of all possible networks an *exhaustive search *to distinguish it from the GHC method.

#### Step 1: Dividing the whole problem into partial problems

Our approach first divides the set of all genes *V *into all the combinational subset with three genes (triplets) *t = {v*_*i*_, *v*_*j*_, *v*_*k *_∈ *V*|1 ≤ *i *<*j *<*k *≤ 5}. For example, our approach obtains _5_C_3 _= 10 partial problems {*v*_1_, *v*_2_, *v*_3_}.{*v*_1_, *v*_2_, *v*_4_}, ..., {*v*_3_, *v*_4_, *v*_5_}.

#### Step 2: Estimating gene regulatory networks

After making partial problems, we next calculate independently the scores of all the possible networks of each partial problem by exhaustive search and obtain estimated DAGs *G*. The number of possible alternative networks for a triplet *{v*_1_, *v*_2_, *v*_3_*} *is 3^3 ^= 27 because there are three cases for each potential edge (*v*_*i*_, *v*_*j*_) (1 ≤ *i *<*j *≤ 3): a directed edge from *v*_*i *_to *v*_*j *_, a directed edge from *v*_*j *_to *v*_*i*_, and no edge.

Let *c *= (*D*, *S*_*D*_, *R*_*D*_) be a tuple, where *D *∈ *G *is a DAG, *S*_*D *_*= p*(*D, d*) is a score of *D*, where *p*(*D, d*) is given by Equation 2, and *R*_*D *_is a rank of *D*.

We add tuples of all the partial problems to *Z*, where *Z *is a set of *c*. For example, when we have 10 partial problems {*v*_1_, *v*_2_, *v*_3_}.{*v*_1_, *v*_2_, *v*_4_}, ... , {*v*_3_, *v*_4_, *v*_5_}, we add 270 tuples of networks to *Z*.

#### Step 3: Uniting estimated partial problems

To solve the original problem, this step unites three-gene networks into a single gene regulatory network. The policy of the step is to classify relationships between genes, i.e., determine (*v*_*i*_, *v*_*j*_) (1 ≤ *i *<*j *≤ 3) into one of the three edge types (a directed edge from *v*_*i *_to *v*_*j*_, a directed edge from *v*_*j *_to *v*_*i*_, or no edge between *v*_*i *_and *v*_*j*_) according to the score calculated in Step 2.

To select an edge type between genes *v*_*i *_and *v*_*j*_, we calculate an edge (*v*_*i*_, *v*_*j*_) value for each of the three types *t *using the following:

(4) $$\underset{\left(D,S_{D},1\right)\in Z}{\sum }S_{D},$$

where *D *has edge (*v*_*i*_, *v*_*j*_). Then we select one edge type that has the highest total value.

When two or more edge types have the highest total value, we use edge scores of the partial problems whose ranks are 2 or more.

#### Algorithm

**Input**: *V *= *V*_1_, ..., *Vn*: a set of genes, GEP: gene expression profiles of *V*

**Output**: *G*_*V *_: DAG including genes *V*

**Variable**:   *Z*: a set of tuples (graph, score, rank)

1: Make a collection of set **V **that includes all the subsets of *V *with three elements

2-1: for each *U *in **V **do

2-2:   Make a collection of set **D**_**u **_that includes all the DAGs of *U*

2-3:   for each *D *in **D**_**u **_do

2-4:      calculate rank *R*_*D *_and score *S*_*D *_with GEP

2-5:      add (*D*, *S*_*D*_, *R*_*D*_) to *Z*

2-6:   end for

2-7: end for

3-1: *i *← 1

3-2: repeat

3-3:   for each edge between genes (x, y) in D of (*D*, *S*_*D*_, *i*) do

3-4:      add all *S*_*D *_of (*D*, *S*_*D*_, *i*) for each of the three edge types

3-5:      if one edge type has the highest total *S*_*D *_then

3-6:         add an edge between genes (x, y) to *G*_*V*_

3-7:      end if

3-8:      if two or more edge types have the highest total *S*_*D *_then

3-9:         for each edge between genes (x or y, w) in *G*_*V *_, where w is a gene ≠ *x*, *y *do

3-10:            select edge between genes (x, y) from *D *of (*D*, *S*_*D*_, *i*), where *D *includes genes x, y, and w.

3-11:         end for

3-12:         add edge (x, y) selected in (3-10) with the highest *S*_*D *_to *G*_*V*_

3-13:      end if

3-14:   end for

3-15:   *i*←*i*+1

3-16: until directions of all edges in *G*_*V *_are assigned

3-17: return *G*_*V*_

**Figure 2:**
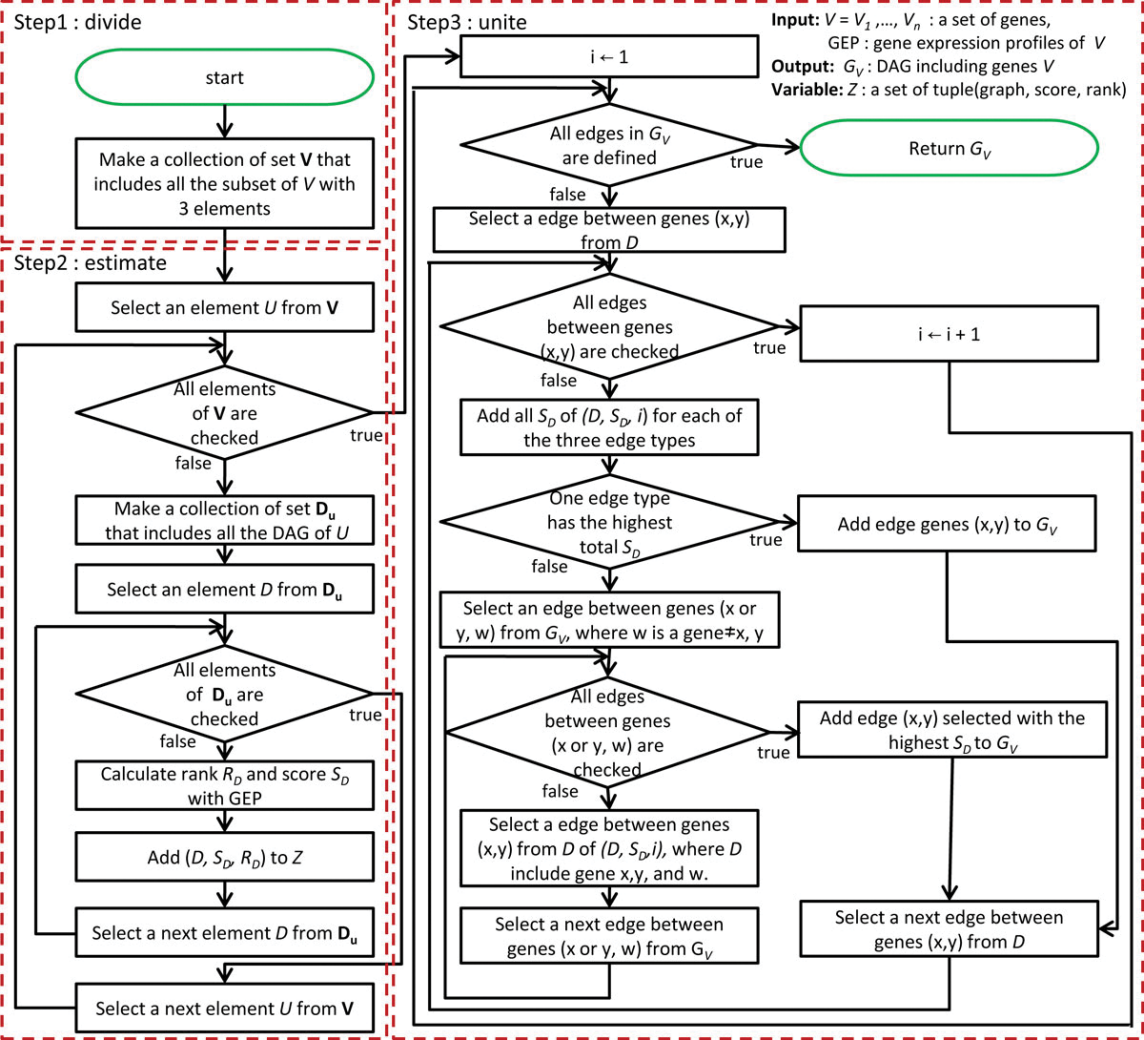
**Flowchart of the algorithm.** Circles represent start and end points. Rectangles represent generic processing steps. Diamonds represent decision steps.

A flowchart of the algorithm can be found in Figure 2.

### Computational experiments

To verify the effectiveness of the proposed method, we performed three experiments. The first experiment determines computational time for different numbers of genes. The purpose of this experiment is to verify that the proposed method is able to estimate gene regulatory networks that are as large-scale as those estimated by the GHC method. The second experiment demonstrates that the proposed method is more accurate than the GHC method. The third experiment shows, through an example, that our algorithm works well for inferring real gene regulatory networks. We estimate the networks, including the known gene regulatory network, and compare the network estimated by the proposed method and that by the GHC method.

#### Implementation, system, and materials

Steps 1 and 2 are implemented using the deal package version 1.2-33 written in R. We use R 2.10.1. Step 3 is implemented using Perl 5.10.1.

The GHC method is implemented in the deal package version 1.2-33. In these experiments, the maximum number of actions, i.e., adding, deleting, or reversing a directed edge, is set at 50 and the number of restarts is set at 0. We call these parameters the default parameter set.

We performed all the experiments on a computer with Intel Core2 Duo 6600 CPU 2.40 GHz processors with 3.0 GB memory. The operation system is Ubuntu 10.04.

We used a dataset of two time-series gene expression profiles including 45102 genes from a mouse adipocyte and osteoblast. The number of time points is 62.

##### Experiment 1

**Figure 3:**
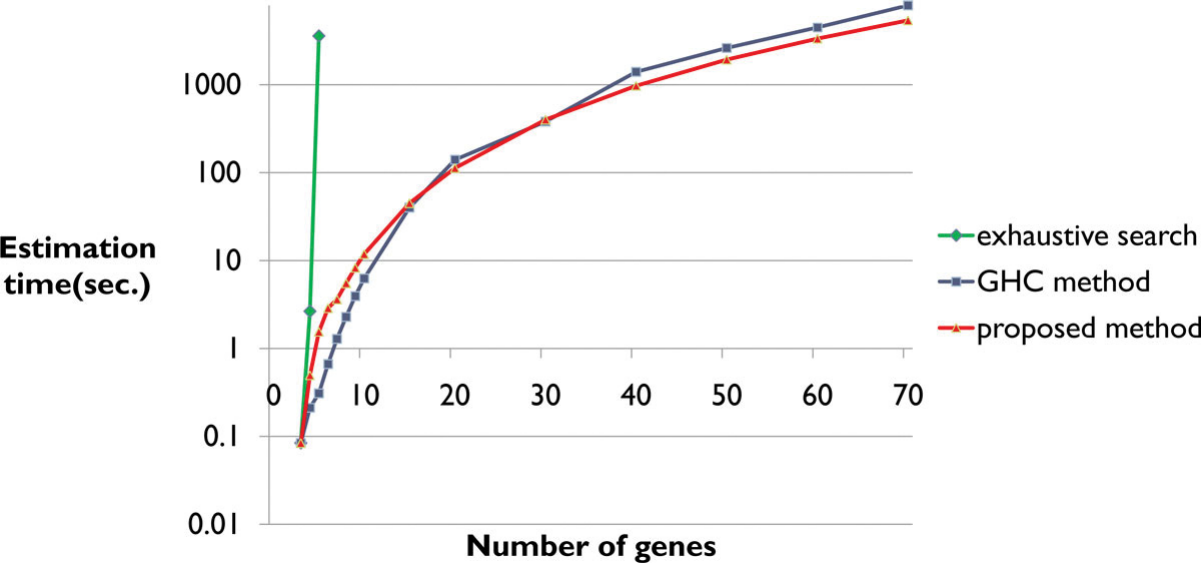
**Comparison of the estimation time.** The estimation time of the exhaustive search, the GHC method, and the proposed method.

We verified that the proposed method can estimate gene regulatory networks as large-scale as those estimated by the GHC method. We used the proposed method, an exhaustive search, and the GHC method, and compared the estimation time for from 3 to 70 genes. In this experiment, we selected genes from the gene expression profile from a mouse adipocyte by random sampling. We ran this process 50 times and calculated the mean estimation time. The results are summarized in Figure 3.

In Figure 3, the horizontal axis corresponds to the number of genes and the vertical axis corresponds to the logarithm of the estimation time. The proposed method was able to estimate the network including 70 genes, and the estimation times were almost the same as those of the GHC method. The estimation time of the proposed method was shorter than that of the GHC method for 40 or more genes. The estimation time of the proposed method was longer than that of the GHC method for 15 or fewer genes. The estimation time of the exhaustive search was very large by 5 genes.

##### Experiment 2

**Figure 4:**
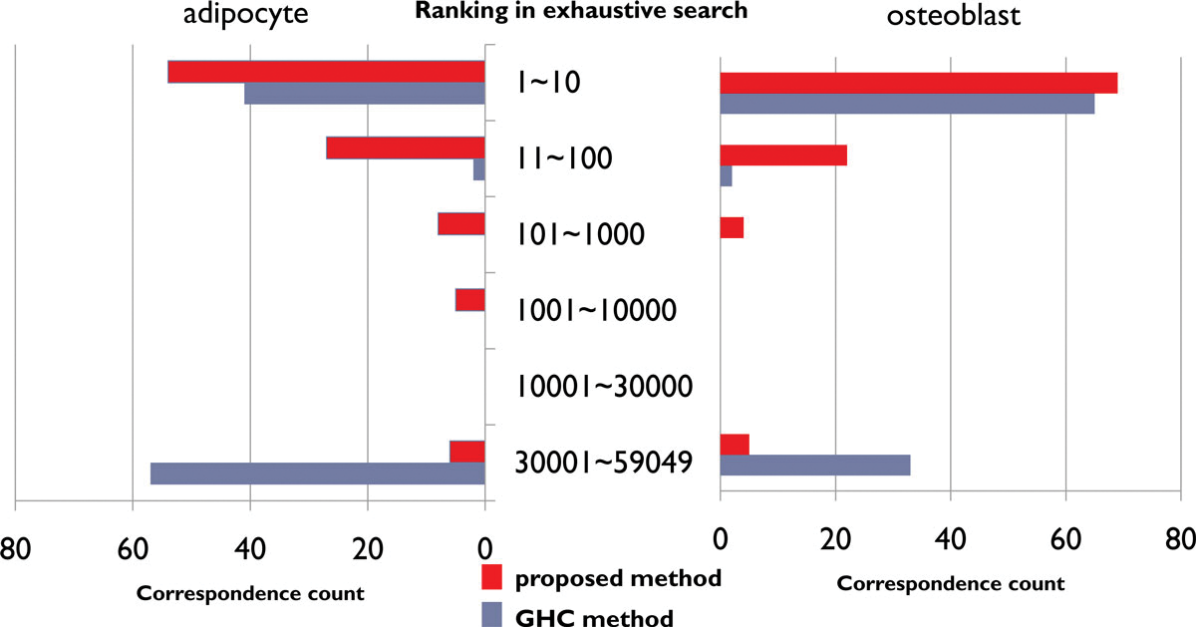
**Comparison of the estimated network.** Frequency that the networks estimated by the GHC method and the proposed method correspond to those of the exhaustive search (from 1 to 59049).

We verified that the estimation accuracy of the proposed method is higher than that of the GHC method for nearly identical estimation times. We compared the estimation results of the exhaustive search with the results of the proposed method and the GHC method. In this experiment, we selected five genes randomly from the gene expression profile 100 times from a mouse adipocyte and osteoblast. We estimated the network of these five genes by the proposed method and the GHC method. There are 59049 DAGs for five genes, and all the DAGs are ranked by the scores of the exhaustive search. The ranking was used to evaluate the networks estimated by the proposed method and the GHC method. The results are listed in Figure 4.

The two bar charts in Figure 4 show the ranks of 100 networks estimated by the proposed method and the GHC method. The left bar chart is the results for adipocyte, and the right are those for osteoblast. The correspondence count is the number of times that the network estimated by the proposed method or the GHC method corresponded with the network of the exhaustive search. The ranking in the exhaustive search is the ranking of the networks estimated by the exhaustive search. The networks are ranked by the scores of the exhaustive search. As there are 59049 DAGs for five nodes, the ranks are from 1st to 59049th.

The correspondence count of the proposed method from the 1st to 10th networks of the exhaustive search exceeded 50. For the correspondence count from the 30001th to the 59049th network of the exhaustive search, the GHC method exceeded 50 and the proposed method was less than 10.

##### Experiment 3

**Figure 5:**
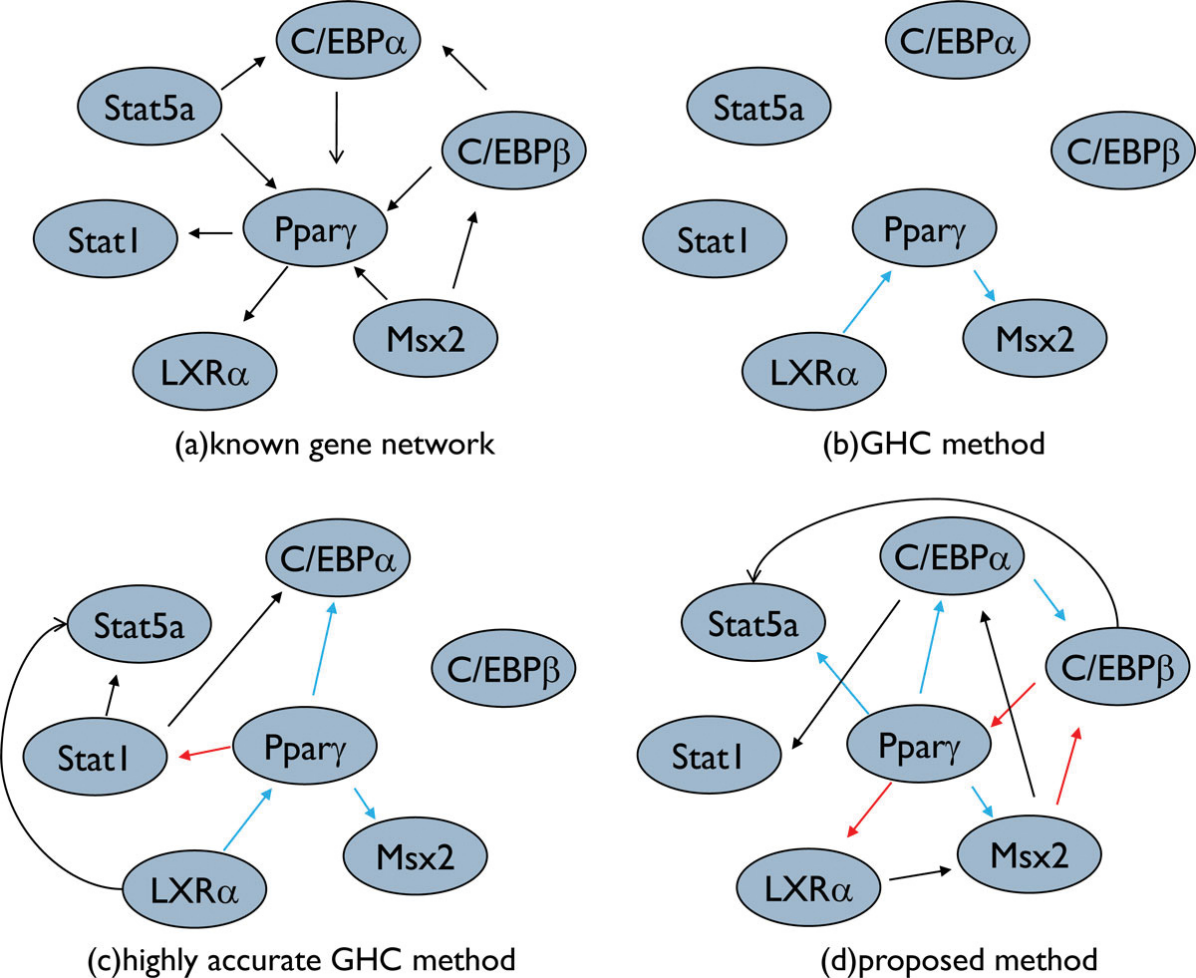
**Comparison of the network including *Ppar*γ and genes that regulate or are regulated by *Pparγ.*** (a) is the known gene regulatory network. (b) is the network estimated by the GHC method with the maximum number of actions set at 50 and the number of restarts set at 0. (c) is the network estimated by the GHC method with the maximum number of actions set at 100 and the number of restarts set at 10. (d) is the network estimated by the proposed method. Blue circles represent genes. Red edges indicate edges also in network (a), blue edges indicate edges with a different direction from those in network (a), and black edges indicate that there are no such relationships in network (a).

We used a known gene regulatory network and verified that the proposed method can estimate more accurately than the GHC method with the same or less computational time. We compared the regulations estimated by the proposed method with those of the GHC method. In this experiment, we used 40 genes from the gene expression profile from a mouse adipocyte. Of these, 7 genes are *Pparγ *and the genes that regulate or are regulated by *Pparγ *in adipocyte. These are shown in Figure 5(a). The remaining 33 genes were selected by random sampling. The results and known networks are shown in Figure 5. In this experiment, we used two parameter sets for the GHC method. One is the default parameter set. In the other parameter set, the maximum number of actions is 100 and the number of restarts is 10, which will return a better network but requires about 20-fold longer computational time than the default.

In Figure 5, results of the default and other parameter set are shown as networks (b) and (c), respectively. We call (c) the network estimated by the highly accurate GHC method in this experiment. Network (d) is estimated by the proposed method. The edges in networks (b), (c), and (d) are categorized according to the edges of network (a). The red edges are also in network (a), the blue edges have a different direction from those in network (a), and the black edges have no relationship in network (a).

Figure 5 shows that the proposed method was able to estimate more correctly than the GHC method. The *sensitivity *and *selectivity *of the proposed method were 33% and 30%, those of the GHC method were 0% and 0%, and those of the high accurate GHC method were 11% and 14%. Networks (b), (c), and (d) have many edges that the known gene regulatory network does not have, but these edges describe indirect regulations. For example, in Figure 5(d), there is a black edge from *C/EBPα *to *Stat*1. The edge describes the indirect regulation from *C/EBPα *to *Stat*1 via *Pparγ *because there are edges from *C/EBPα *to *Pparγ *and from *Pparγ *to *Stat*1 in Figure 5(a).

## Discussion

The GHC method tends to produce local optimal solutions. For example, in Figure 4, the results of the GHC method have two peaks, corresponding to the classes of 1-10 and 30001-59049. We cannot completely avoid selecting a local optimal solution when using the GHC method, because the solution accuracy depends on the initial DAG from which the search is started. To obtain the best network when using the GHC method, the estimation must be repeated using different initial DAGs. In contrast, the proposed method can produce one result as the best network.

The results of our experiments indicate that dividing the set of all genes and uniting the network results can estimate more accurately than the GHC method. With the GHC method, the maximum number of actions, i.e., adding, deleting, or reversing a directed edge, and the number of restarts can be adjusted. If these parameters are increased as much as possible, the estimation accuracy can be made comparable to that of the exhaustive search. However, this would spoil the advantage of the GHC method that it can estimate with high speed. The GHC method selects the action that increases the network score the most; therefore, a regulation that increases the network score only slightly is rarely selected. In this sense, the search of the GHC method is considerably biased. This aspect becomes pronounced when the limiting parameters are set strictly. With the proposed method, regulations that have a positive effect will be selected independently of whether that effect is slight or strong. For example, in Figure 5, the regulatory relationship between *Pparγ *and *C/EBPβ *could not be estimated by the GHC method, even if the parameters of the restart and the actions were significantly increased.

We verified that the proposed method can estimate networks as large-scale as those estimated using the GHC method. We spend at most 0.1 second to estimate the network of one partial problem with three genes and repeat the estimation _*n*_*C*_3 _times in the proposed method. Therefore, the proposed method can estimate the network with a low amount of memory compared with the GHC method, which, like the exhaustive search, requires much memory. When we estimate a network for a data set from a large number of genes using the GHC method, it is easy to run out of memory, making the actual computational time longer than the theoretical time.

## Conclusions

In this study, we present a novel BN-based deterministic method with reduced computational time. We confirmed experimentally that the proposed method can reduce the computational time drastically without degrading the solution accuracy. The proposed method can estimate networks as large-scale as those estimated by the GHC method. Furthermore, the proposed method can estimate more accurately than the GHC method, even if the computational time of the GHC method is increased to more than 20 times that of the proposed method.

## Competing interests

The authors declare that they have no competing interests.

## Authors' contributions

YW implemented the algorithm and performed the analyses. YW, SS, YT, and HM conceived and designed the experiments and wrote the paper.
